# Nonprescription Hormone Use Among Transgender Women **—** National HIV Behavioral Surveillance Among Transgender Women, Seven Urban Areas, United States, 2019***–***2020

**DOI:** 10.15585/mmwr.su7301a4

**Published:** 2024-01-25

**Authors:** Evelyn Olansky, Kathryn Lee, Senad Handanagic, Lindsay Trujillo, National HIV Behavioral Surveillance among Transgender Women Study Group, National HIV Behavioral Surveillance Among Transgender Women Study Group, Narquis Barak, Kathleen A. Brady, Sarah Braunstein, Jasmine Davis, Sara Glick, Andrea Harrington, Jasmine Lopez, Yingbo Ma, Aleks Martin, Genetha Mustaafaa, Tanner Nassau, Gia Olaes, Jennifer Reuer, Alexis Rivera, William T. Robinson, Ekow Kwa Sey, Sofia Sicro, Brittany Taylor, Dillon Trujillo, Erin Wilson, Pascale Wortley

**Affiliations:** ^1^Social & Scientific Systems, Inc., Silver Spring, Maryland; ^2^ICF, Fairfax, Virginia; ^3^Division of HIV Prevention, National Center for HIV, Viral Hepatitis, STD, and TB Prevention, CDC, Atlanta, Georgia; CrescentCare; Philadelphia Department of Public Health; New York City Department of Health and Mental Hygiene; CrescentCare; University of Washington, School of Medicine, Division of Allergy and Infectious Diseases; Public Health – Seattle & King County, HIV/STD Program; Philadelphia Department of Public Health; New York City Department of Health and Mental Hygiene; Los Angeles County Department of Public Health; Public Health – Seattle & King County, HIV/STD Program; Georgia Department of Public Health; Philadelphia Department of Public Health; Los Angeles County Department of Public Health; Washington State Department of Health; New York City Department of Health and Mental Hygiene; Louisiana State University Health Science Center in New Orleans – School of Public Health; Louisiana Office of Public Health STD/HIV/Hepatitis Program; Los Angeles County Department of Public Health; San Francisco Department of Public Health; Georgia Department of Public Health; San Francisco Department of Public Health; San Francisco Department of Public Health; Georgia Department of Public Health.

## Abstract

Certain transgender women who seek gender-affirming hormone treatment (GAHT) face economic and social barriers that limit or prevent access to medically supervised GAHT. Transgender women facing such barriers might acquire GAHT without prescription, potentially without proper dosage, administration, and health monitoring in the absence of medical supervision. For this report, survey data were analyzed from 1,165 transgender women in seven urban areas in the United States to examine associations between self-reported use of nonprescription GAHT and known correlates of nonprescription GAHT, including cost, insurance coverage for GAHT, homelessness, receiving money or drugs in exchange for sex during the past 12 months (exchange sex), lack of comfort discussing gender with provider, and lack of health care use. After controlling for complex sampling design, transgender women who reported recent health care use or insurance coverage for GAHT were less likely to report nonprescription GAHT, and those reporting recent exchange sex or recent homelessness were more likely to report nonprescription GAHT. Findings suggest that transgender women were more likely to use GAHT without a prescription in situations of economic and social marginalization (e.g., disengagement from health care, lack of insurance or trans-specific health care, homelessness, or engagement in sex work). Public health professionals can use these results to design effective interventions to facilitate prescribed hormone use among transgender women in the United States, although access to housing, trans-affirming health care, and insurance coverage might be needed to prevent nonprescription use.

## Introduction

Certain transgender women receive or desire gender-affirming hormone treatment (GAHT) (*1*), initiation of which is associated with higher quality of life (*2*,*3*), decreased depression (*3*), and reduced HIV treatment interruptions (*4*) among transgender women. Although hormone treatment is known to be safe and effective when obtained from a health care professional (*5*), certain transgender women use hormones without a prescription (*1*,*6*,*7*). Although masculinizing GAHT is a Schedule III controlled substance, feminizing GAHT is not a scheduled or controlled substance, but it is not approved by the Food and Drug Administration for over-the-counter use, leaving those seeking non-prescription feminizing GAHT uncertain if what they are doing is punishable by law. Side effects and health risks associated with GAHT among transgender women include ischemic heart disease and hypertension (*8*). Although GAHT prescribers can monitor and manage potential side effects among their patients, transgender women who use nonprescription hormones might be unaware of the potential side effects, unaware of proper dosage, or unable to prevent or manage adverse effects. Because of the potential risks, it is important to understand potential factors associated with nonprescription hormone use and identify possible barriers to safe, medically monitored GAHT. Such data can be used to guide development of interventions or policy changes that reduce nonprescription use of hormones among transgender women.

Among convenience samples of transgender women, correlates of nonprescription hormone use are typically based on studies in a single state, city, or clinical population. Although limited, previous studies suggest that nonprescription hormone use is correlated with lacking health insurance (*1*), experiencing homelessness (*9*) and a history of receiving money or drugs in exchange for sex during the past 12 months (hereafter, exchange sex) (*10*). These findings suggest that where nonprescription hormone use occurs, barriers to needed resources (e.g., housing and licit income) and barriers to affordable health care services are more likely to be present. This analysis explored demographic, health care, and economic correlates of nonprescription hormone use among transgender women in seven urban areas in the United States who participated in CDC’s National HIV Behavioral Surveillance Among Transgender Women (NHBS-Trans). Understanding the correlates of nonprescription hormone use, particularly those within the purview of public health policy, such as health insurance coverage and health care access, might facilitate prescribed use of GAHT and prevent the risks associated with nonprescription GAHT use. Conversely, recent nonprescription GAHT use might be a useful indicator of barriers to health care among transgender women who use or desire to use hormones.

## Methods

### Data Source

This report includes survey data from NHBS-Trans conducted by CDC during June 2019***–***February 2020 to assess behavioral risk factors, PrEP, antiretroviral therapy, condom use, and HIV prevalence. Eligible participants completed an interviewer-administered questionnaire and were offered HIV testing. Additional information about NHBS-Trans eligibility criteria, data collection, and biologic testing is available in the overview and methodology report of this supplement (*11*). The NHBS-Trans protocol questionnaire and documentation are available at https://www.cdc.gov/hiv/statistics/systems/nhbs/methods-questionnaires.html#trans. 

Applicable local institutional review boards in each participating project area approved NHBS-Trans activities. The final NHBS-Trans sample included 1,608 transgender women in seven urban areas in the United States (Atlanta, Georgia; Los Angeles, California; New Orleans, Louisiana; New York, New York; Philadelphia, Pennsylvania; San Francisco, California; and Seattle, Washington) recruited by using respondent-driven sampling (RDS) This activity was reviewed by CDC, deemed not research, and was conducted consistent with applicable federal law and CDC policy.*

Participants were included in this analysis if they reported current or recent (during the past 12 months) hormone use and provided responses to questions about age, poverty, network size, insurance coverage, and access to transgender-specific health care (n = 1,165). The analysis excluded participants who did not use hormones during the past 12 months (the recall period for nonprescription hormone use and other NHBS-Trans measures) to avoid conflating participants with insurmountable barriers to hormone use and those not using hormones for other reasons. 

### Measures

Assessed demographic characteristics included age group (18*–*29, 30*–*39, 40*–*49, and ≥50 years) and education (less than high school, high school, some college or technical degree, and college degree or more). Other characteristics assessed included transgender-specific health insurance coverage, transgender-specific health care (as measured by ever having a health care provider with whom they felt comfortable discussing gender-related health issues), current hormone use, hormone use during the past 12 months, visiting a health care provider during the past 12 months, current or recent homelessness, recent sex work, and use of nonprescription hormones during the past 12 months (Table 1).

**TABLE 1:** Variables, measures, and analytic coding — National HIV Behavioral Surveillance Among Transgender Women, seven urban areas,* United States, 2019*–*2020

Variable	Question	Analytic coding
Transgender-specific health insurance coverage	Do you currently have health insurance or health care coverage?	Yes (current health insurance coverage covers hormones for gender transition or affirmation) or no (no current health insurance coverage, or current health insurance coverage does not cover hormones for gender transition or affirmation)
	Does your current health insurance cover hormones for gender transition or affirmation?	
Transgender-specific health care	Do you have a health care provider with whom you feel comfortable discussing gender-related health issues? Have you ever had a health care provider with whom you felt comfortable discussing gender-related health issues?	Yes (currently or ever had a health care provider with whom you feel comfortable discussing gender-related health issues) or no (Have never had a health care provider with whom you feel comfortable discussing gender-related health issues).
Current hormone use	Are you currently taking hormones for gender transition or affirmation?	Yes or no (among those reporting lifetime hormone use)
Recent hormone use	In the past 12 months, have you used hormones for gender transition or affirmation?	Yes or no (among those reporting lifetime hormone use)
Recent nonprescription hormone use	In the past 12 months, have you used hormones that were not prescribed to you by a doctor or other health care professional?	Yes or No (among those reporting recent or current hormone use)

* Atlanta, GA; Los Angeles, CA; New Orleans, LA; New York City, NY; Philadelphia, PA; San Francisco, CA; and Seattle, WA.

### Analysis

Log-linked Poisson regression with generalized estimating equations were used to examine the association between nonprescription hormone use and social and structural factors related to health care access. Bivariate Poisson regression was performed to identify factors associated with recent use of nonprescription hormones. Respondent-driven sampling methodology and network effects were accounted for by clustering on recruitment chain, urban area, and self-reported network size; results are reported as adjusted prevalence ratios with 95% CIs. Variables significant (p<0.05) in bivariate analyses were carried forward to a multivariate model, and variables that remained significant (p<0.05) in multivariate analysis were included in the final model. Analyses were performed using SAS software (version 9.4; SAS Institute).

## Results

Among the 1,165 transgender women who reported any hormone use during the past 12 months, 91.7% reported current hormone use. Among transgender women who used hormones during the past 12 months, transgender women aged 40***–***49 years (9.7%) and aged ≥50 years (16.1%) were significantly less likely than transgender women aged 18***–***29 years (22.8%) to report nonprescription hormone use (Table 2). Transgender women not recently visiting a health care provider (47.1%), lacking transgender-specific health insurance coverage (38.4%), lacking access to transgender-specific health care (36.0%), and not currently using hormones (35.1%) were more likely to report nonprescription hormone use compared with transgender women who had not experienced these health care challenges. Transgender women who experienced current or recent homelessness were significantly more likely to report nonprescription hormone use (27.6%) than those who had not experienced homelessness (13.9%). Transgender women who reported recent exchange sex were significantly more likely to report nonprescription hormone use (28.8%) than those who did not report recent exchange sex (15.1%). Despite the observed associations between nonprescription hormone use and economically tenuous circumstances (e.g., homelessness and exchange sex), no significant association was observed between nonprescription hormone use and poverty (at or below the poverty level as measured by the 2019 Federal poverty level). After controlling for age, education, RDS, and urban area, use of nonprescription hormones was more common than use of only prescription hormones among transgender women who did not have transgender-specific health insurance coverage, who did not visit a health care provider recently, who experienced current or recent homelessness, and who reported recent exchange sex (Table 3).

**TABLE 2:** Number and percentage of transgender women receiving nonprescription gender-affirming hormone treatment, by selected characteristics — National HIV Behavioral Surveillance Among Transgender Women, seven urban areas,* United States, 2019*–*2020^†^

Characteristic	Total (N = 1,165)	Recent^§^ nonprescription gender-affirming hormone treatment	Independent association with nonprescription gender-affirming hormone treatment^¶^	
	No.	No. (%)	PR	(95% CI)
**Overall**	**1,165**	**230 (19.7)**	**—**	**—**
**Age group, yrs**				
18*–*29	**364**	83 (22.8)	Ref	**—**
30*–*39	**330**	86 (26.1)	1.15	(0.87***–***1.51)
40*–*49	**228**	22 (9.7)	0.42	(0.31*–*0.58) **
≥50	**242**	39 (16.1)	0.73	(0.54*–*0.98) **
**Education**				
<high school	**244**	47 (19.3)	Ref	**—**
High school	**439**	91 (20.7)	1.08	(0.84***–***1.39)
Some college or technical degree	**353**	71 (20.1)	1.05	(0.76***–***1.44)
College degree or more	**129**	21 (16.3)	0.88	(0.58***–***1.33)
**Health care access**				
Transgender-specific health insurance coverage**				
Yes	**925**	136 (14.7)	Ref	**—**
No	**211**	81 (38.4)	2.48	(1.98*–*3.11)**
**Current gender-affirming hormone treatment**				
Yes	**1,068**	196 (18.4)	Ref	**—**
No (recent**^†^** but not current)	**97**	34 (35.1)	1.82	(1.50*–*2.20)**
**Visited health care provider recently^§^**				
Yes	**1,114**	206 (18.5)	Ref	**—**
No	**51**	24 (47.1)	2.55	(2.10*–*3.10)**
**Transgender-specific health care** ^††^				
Yes	**1,054**	190 (18.0)	Ref	**—**
No	**111**	40 (36.0)	1.91	(1.2*–*2.83)**
**Economic circumstances**				
**Poverty^§§^**				
Above Federal poverty level	**417**	86 (20.6)	Ref	**—**
At or below Federal poverty level	**740**	142 (19.2)	0.91	(0.69***–***1.20)
**Experienced homelessness** ^¶¶^				
Currently or recently**^§^** homeless	**496**	137 (27.6)	1.96	(1.59*–*2.41)**
Not currently or recently**^§^** homeless	**669**	93 (13.9)	Ref	**—**
Recent^§^ exchange sex***				
Yes	**400**	115 (28.8)	1.88	(1.48*–*2.38)**
No	**764**	115 (15.1)	Ref	**—**

**Abbreviations:** PR = prevalence ratio; Ref = referent group.

* Atlanta, GA; Los Angeles, CA; New Orleans, LA; New York City, NY; Philadelphia, PA; San Francisco, CA; and Seattle, WA.

^†^ N = 1,165 participants. Numbers might not sum to totals because of missing data.

**^§^** Reported within a reference period of 12 months.

^¶^ Denotes comparisons made using log-linked Poisson regression with generalized estimating equations, adjusted for respondent-driven sampling design, controlling for network cluster and city.

** Statistically significant; 95% CIs do not cross the null of 1.0.

^††^ “Transgender-specific health care” measured as “Ever having a provider with whom you are comfortable discussing gender related issues.”

**^§§^** 2019 Federal poverty level thresholds were calculated on the basis of U.S. Department of Health and Human Services Federal poverty level guidelines (https://aspe.hhs.gov/topics/poverty-economic-mobility/poverty-guidelines/prior-hhs-poverty-guidelines-federal-register-references/2019-poverty-guidelines).

^¶¶^ Homelessness was defined as having lived on the street, in a shelter, in a single room occupancy hotel, or in a car during the past 12 months.

*** Exchange sex was defined as having received money or drugs in exchange for sex during the past 12 months.

**TABLE 3:** Associations with nonprescription gender-affirming hormone treatment among transgender women* — National HIV Behavioral Surveillance Among Transgender Women, seven urban areas,^†^ United States, 2019*–*2020

Variable	aPR	95% CI
Health care access		
Transgender-specific health insurance coverage		
Yes	Ref	**—**
**No**	2.26	(1.80*–*2.84)^§^
**Visited health care provider recently** ^¶^		
Yes	Ref	**—**
No	**1.81**	**(1.45*–*2.27)^§^**
Economic conditions		
Experienced homelessness**		
**Yes**	**Ref**	**—**
No	**1.76**	**(1.43*–*2.19)^§^**
Recent exchange sex^††^		
**No**	**Ref**	**—**
Yes	**1.62**	**(1.30*–*2.01)^§^**

**Abbreviations:** aPR = adjusted prevalence ratio; Ref = referent group.

* Denotes comparisons made using log-linked Poisson regression with generalized estimating equations, adjusted for respondent-driven sampling design, controlling for network cluster and city.

**^†^** Atlanta, GA; Los Angeles, CA; New Orleans, LA; New York City, NY; Philadelphia, PA; San Francisco, CA; and Seattle, WA.

^§^ Statistically significant; 95% CIs do not cross the null of 1.0.

^¶^ Reported within a reference period of 12 months.

** Homelessness was defined as having lived on the street, in a shelter, in a single room occupancy hotel, or in a car during the past 12 months.

**^††^** Exchange sex was defined as having received money or drugs in exchange for sex during the past 12 months.

## Discussion

Among transgender women who reported current or recent use of hormones, nonprescription hormone use was associated with conditions of economic hardship (e.g., current or recent experience of homelessness or sex work) and limited access to health care (e.g., lacking health insurance coverage for hormonal treatment and not visiting a health care provider during the past 12 months). Transgender women who used hormones without transgender-specific health insurance coverage were more than twice as likely to use nonprescription hormones than transgender women who used hormones and had transgender-specific health insurance coverage. Transgender women whose engagement in health care is infrequent, such as among those who have not received medical care in more than 12 months, were also significantly more likely to report use of nonprescription hormones. Transgender women aged >40 years were significantly less likely than transgender women aged 18–29 years to report use of nonprescription hormones, although those differences were not significant when controlling for covariates.

Although economic marginalization appears to be the primary driver for use of nonprescription hormones, the association might be influenced by additional factors. Notably, no association was observed between use of nonprescription hormones and reported income below the 2019 Federal poverty level. A lack of transgender-specific health insurance coverage or a lack of recent engagement with a health care provider poses clear logistical barriers to accessing prescribed hormones because it is difficult to maintain a prescription without engaging with a provider and expensive to maintain a prescription that is not covered by health insurance. Although the findings in this report suggest that transgender women recently experiencing homelessness or exchange sex face many barriers to accessing prescription hormones, the nature of those barriers might be more complex and difficult to interpret than barriers such as lack of insurance coverage and lack of recent health care engagement. Challenges associated with experiencing homelessness, such as difficulty contacting and physically accessing health care (*12*), or challenges associated with sex exchange, such as fear of discrimination or stigma in health care settings (*13*), might explain the observed associations between prescription and nonprescription hormone use by experiencing homelessness or sex work. Future studies should examine additional factors in health care access that might be mediated by experiencing homelessness or exchange sex, such as access to transportation, methods of contacting providers, or anticipated stigma.

Despite previous findings suggesting that medical mistrust and anticipated mistreatment are key factors for transgender persons who are not actively engaged in health care (*14*), lacking transgender-specific health care was not significantly associated with use of nonprescription hormones in the final model. A multi-item measure of medical mistrust and further investigation of each respondent’s rationale for using nonprescription hormones could provide valuable insight into how best to prevent nonprescription use. In this sample and survey, the use of nonprescription hormones versus prescription hormones appears to be associated primarily with economically tenuous circumstances (e.g., participating in exchange sex or experiencing homelessness), despite not being directly associated with poverty as determined by the 2019 Federal poverty level. When appropriately administered, hormone treatment can cost hundreds of dollars to initiate even when insured and might come from a culturally insensitive provider. As a result, many transgender persons choose to independently obtain and administer their own hormone treatment. Even if the dangers of using nonprescription hormones are known, the dangers of stigmatizing and unsupportive experiences in health care and high medical costs might be more salient for those who desire hormonal treatment.

## Limitations

General limitations for NHBS-Trans are available in the overview and methodology report of this supplement (*11*). The findings in this report are subject to at least six additional limitations. First, the results are not representative of transgender women residing outside the seven urban areas. Because transgender women are hard to reach, the data might not be representative of all transgender women residing in the seven urban areas. However, this is the first time behavioral and contextual data were successfully collected through systematic biobehavioral surveillance of transgender women. Second, all data used for this analysis were self-reported and are subject to recall and social desirability biases, particularly concerning sex behavior, exchange sex and drug use (*15*). Social desirability could bias the results in multiple ways, whether through underreporting of nonprescribed GAHT use because participants are not certain whether it is punishable by law or underreporting of correlates such as homelessness or exchange sex because of social stigma and perceived legal consequences. Third, analyses were limited to cross-sectional associations; therefore, temporality or causation could not be assessed. Fourth, the survey did not collect reasons for using nonprescription hormones, for never taking hormones, or for stopping hormone treatment, all of which could help identify the primary barriers to obtaining hormones via prescription. Fifth, measures of access to transgender-specific health care and insurance coverage used in this survey were minimal, each focusing on a single criterion (comfort discussing transgender health with their provider and insurance coverage specifically for hormone treatment, respectively). Willingness to discuss transgender-specific health with a provider does not imply the care received was appropriate or culturally competent, so this measure might better reflect a scarcity of affirming providers than it reflects availability of quality care. Similarly, although insurance coverage for hormones is an essential part of transgender health care, the survey did not assess if respondents had insurance coverage for the specific forms of hormone treatment they desire, or coverage for the many other forms of gender-affirming care often sought by transgender persons. Finally, the question assessing homelessness is limited and doesn’t include transitory instances of housing instability (e.g., couch surfing).

## Conclusion

This analysis is the first to examine use of nonprescription hormones among transgender women in a sample from multiple urban areas. The geographical breadth and rigorous sampling strategy reinforce findings from previous research, further demonstrating association between use of nonprescription hormones compared with only prescription hormones and clear indicators of social and economic marginalization, such as disengagement from health care (*6*,*7*), lack of adequate insurance coverage (*1*), recent participation in exchange sex (*10*), and experiencing recent homelessness (*9*).

An important topic for future research is understanding reasons for using nonprescription hormones. GAHT is regarded as essential health care for transgender persons who desire it, a determination supported by extensive research demonstrating the positive health outcomes associated with hormone therapy, as well as reduction of negative health outcomes associated with hormone therapy (*2*,*16*). Where gender-affirming hormones are desired but not accessible through safe and legal means, nonprescription sources provide an alternative means of access. If the conditions that necessitate nonprescription hormone use can be mitigated, whether through tailored social programs to provide accessible and affordable hormone treatment administered by gender-affirming providers or through broader actions to prevent economic disadvantage among transgender persons, nonprescription hormone use can be abandoned in favor of more accessible treatment under appropriate medical supervision.

## Footnotes

* 45 C.F.R. part 46.102(l)(2), 21 C.F.R. part 56; 42 U.S.C. Sect. 241(d); 5 U.S.C. Sect. 552a; 44 U.S.C. Sect. 3501 et seq.
